# Role of Anti-Müllerian Hormone in the Pathogenesis of Polycystic Ovary Syndrome

**DOI:** 10.3389/fendo.2020.00641

**Published:** 2020-09-09

**Authors:** Didier Dewailly, Anne-Laure Barbotin, Agathe Dumont, Sophie Catteau-Jonard, Geoffroy Robin

**Affiliations:** ^1^Inserm, Laboratory of Development and Plasticity of the Neuroendocrine Brain, Jean-Pierre Aubert Research Centre, Lille, France; ^2^CHU Lille, Institut de Biologie de la Reproduction-Spermiologie-CECOS, Hôpital Jeanne de Flandre, Lille, France; ^3^CHU Lille, Unité Fonctionnelle de Gynécologie Endocrinienne, Service de Gynécologie Médicale, Orthogénie et Sexologie, Hôpital Jeanne de Flandre, Lille, France; ^4^CHU Lille, Service d'Assistance Médicale à la Procréation et Préservation de la Fertilité, Hôpital Jeanne de Flandre, Lille, France; ^5^Lille University, EA 4308 “Gametogenesis and Gamete Quality”, Lille, France

**Keywords:** anti-müllerian hormone, polycystic ovary syndrome, androgens, follicle, FSH, anovulation, GnRH, aromatase

## Abstract

Besides its interest for diagnosis, the finding of an elevated serum AMH level in PCOS has open major pathophysiological issues. This review addresses the three most important issues: 1- the role of AMH in the disturbed folliculogenesis of PCOS; 2- the role of AMH in the gonadotropin dysregulation of PCOS and 3- the role of AMH in the trans-generational transmission of PCOS. For each of those issues, the clinical and experimental evidences currently available are discussed and pathophysiological hypothesis are proposed.

## Introduction

Polycystic ovarian syndrome (PCOS) is the most common endocrine disorder in women of childbearing age and the leading cause of hyperandrogenism (HA) and oligo-anovulation (OA) causing infertility (1). PCOS is characterized by an increased number of ovarian follicles at all growing stages (2–4). This increase is particularly seen in the pre-antral and small antral follicles. Interestingly, it is precisely those follicles that primarily produce AMH (5, 6). Release of AMH from the granulosa cells (GCs) of antral follicles leads to measurable serum levels, and these concentrations have shown to be proportional to the number of developing follicles in the ovaries. The development of sensitive assays has enabled measuring AMH in serum and its level was found 2–4 fold higher in women with PCOS than in healthy women, as detailed in other articles of this series. This elevated serum AMH level was initially considered a reflection of the increased stock of pre-antral and small antral follicles within polycystic ovaries (PCO) (7, 8). In addition, it could also result from an increased production of AMH per follicle (9), due to an intrinsic property of GCs in PCO that will be discussed below.

The elevated serum AMH level in PCOS quickly interested PCOS specialists who saw it as a way of circumventing the heterogeneity of the ultrasound description of polycystic ovarian morphology (PCOM) that is used in the definition of PCOS. Indeed, the antral follicular count (AFC) being very dependent on the material used, some authors investigated the diagnostic value of the serum AMH assay as a surrogate for follicle number per ovary (FNPO) [reviewed in (10)]. Finally, marking the excess of antral follicles in women with polycystic ovary syndrome (PCOS), AMH assay may soon replace and/or complete the ultrasound ovarian morphology criterion in the diagnosis of this syndrome (11).

Besides its interest for diagnosis, the finding of an elevated serum AMH level in PCOS has open major pathophysiological issues. First, attention has been drawn to its positive association with hyperandrogenism (HA) (7, 8), whom mechanisms are discussed in this review. Then, studies have shown correlation with the PCOS phenotypes, as defined by the Rotterdam criteria (phenotype A: amenorrhea or oligomenorrhea + HA + PCOM; phenotype B: amenorrhea or oligomenorrhea + HA; phenotype C: HA + PCOM; and phenotype D: amenorrhea or oligomenorrhea + PCOM). The highest serum AMH levels are found in phenotype A (12). Conversely, mean AMH serum levels were found to be lower in hyperandrogenic eumenorrheic patients (phenotype C) compared to those with amenorrhea or oligomenorrhea (13), even if they were not hyperandrogenic (phenotype D) (14). This could mean that the AMH excess is the hallmark of a GCs deregulation that plays a major role in the anovulation of PCOS, besides other contributors such as hyperandrogenism and/or excessive LH secretion and/or hyperinsulinism (15). We will discuss this important issue.

Besides the primary autocrine role of AMH in the deregulation of GCs of PCO, the recent discovery of the AMH receptor in a significant subset of GnRH neurons suggests possible extragonadal effects of AMH on the hypothalamic-pituitary-gonadal axis (16) that might be exacerbated in PCOS. Finally, recent data suggest that AMH could be involved in the epigenetic re-programming that is now believed to be the main mechanism leading to PCOS at puberty and adulthood (17). The goal of this review is also to discuss this exciting issue.

## Role of AMH In The Disturbed Folliculogenesis of PCOS

### Is AMH Overexpressed at the Follicle Level?

The hypothesis of a role for AMH in follicular deregulation of PCOS assumes that the expression of this hormone is exaggerated within each follicle and/or that its signaling pathways are amplified. This is difficult to demonstrate *in vivo* because the excess of growing ovarian follicles (up to the stage of small antral follicles) in women with PCOS (2) is a confounding factor. Indeed, this alone could explain the rise in AMH levels because it is those follicles that physiologically secrete AMH (18). In addition, a close correlation has also been shown between plasma AMH levels and the excess of 2–5 mm of antral follicles on ultrasound (8). Thus, it is accepted that the increase in granulosa “mass” secondary to the excess of growing follicles explains at least in part the excess plasma AMH level in women with PCOS (10, 19, 20).

Another explanation, not excluding the first, could be an excess secretion of AMH intrinsic to the growing follicles of women with PCOS (9, 20, 21). Some authors have reported a significant increase in the AMH/AFC ratio in women with PCOS compared with women with asymptomatic ultrasound PCO and non-PCOS controls (6, 22). This suggests a probable over-expression of AMH by the GCs from antral follicles in PCOS women.

In agreement, Pellatt et al. (23) demonstrated *in vitro* in GCs cultures from oophorectomy specimens that the AMH concentration in the culture media was 4 times higher in normo-ovulatory PCOS women and 75 times higher in anovulatory PCOS women compared with GCs from control women. *In vivo*, Das et al. (24) highlighted that the AMH concentration in follicular fluid of 4 to 8 mm antral follicles, was 5 times higher, outside of any ovarian stimulation setting. Catteau-Jonard et al. (25) demonstrated increased transcription of the AMH gene and its receptor by quantitative RT-PCR on partially luteinized GCs collected during oocyte puncture for *in vitro* fertilization in women with PCOS, compared with control women. This increased transcriptional activity was seen in both selected intermediate-sized (8–13 mm mean diameter) and larger dominant follicles (17–22 mm mean diameter). All these data suggest increased expression of AMH by the GCs of women with PCOS, probably secondary to intrinsic dysfunction of these cells.

Nevertheless, all teams do not validate this hypothesis. Owens et al. (26) found no difference in the transcription of the AMH gene or its receptor in their study which compared the expression of 13 genes by quantitative RT-PCR by GCs from small, unstimulated antral follicles (on ovarian cortex sampled for fertility preservation) and on partially luteinized GCs (in patients benefiting from *in vitro* fertilization) in women with PCOS vs. control women. On the other hand, Dilaver et al. (27) found no basal increase in the expression of AMH transcripts in cultured GCs from PCO compared with normal ovaries. These results should nevertheless be put into perspective, given the low level of AMH expression in cultured human GCs and the small number of cases studied.

Another explanation at the molecular level could be an increased stability of the messenger RNAs resulting from the transcription of the AMH gene in the GCs of PCOS women. Thus, even if the transcription of the AMH gene is not increased, an exaggerated stability of the messenger RNAs could lead to a more marked translational activity and thus to an increase in AMH secretion. The degree of polyadenylation of the 3′-untranslated regions (3′-UTR) of mRNAs coding for AMH could be one of the explanations. However, this hypothesis has not been the subject of any specific study to date.

Finally, the role of certain microRNA which are known to be inhibitors of the translation of messenger RNAs, could also be mentioned. Nevertheless, the only study available to date has not been able to confirm this hypothesis.

### If It Is Real, What Is the Explanation for the Excess Production of AMH by GCs From PCO?

At the molecular level, no abnormalities in the AMH gene that could lead to excess transcription have been reported in PCOS women (28). A whole series of studies point to the responsibility of hyperandrogenism, but controversy persists as to the reality of this effect and its mechanisms, which may be direct or indirect.

*In vivo* data in PCOS patients are contradictory. A possible direct stimulatory effect of androgens on the expression of AMH by GCs was first raised when a positive correlation between serum AMH and androgen concentrations was reported in several series of PCOS women (7, 8, 29–31). However, many confounding factors may play a role, in particular the positive effect of androgens on the number of growing follicles (32) and thus on the “granulosa mass.” Caanen et al. (33) observed that administration of androgens as part of female-to-male transitions induced a significant decrease in AMH levels, but the protocol included the use of a GnRH agonist, which might have confused the results by lowering serum FSH level (see below). Finally, the decrease in serum AMH levels in PCOS patients receiving high-dose cyproterone acetate, a progestin with a potent anti-gonadotropic and peripheral anti-androgenic action, was no greater than under other anti-gonadotropic drugs, such as estrogen-progestin contraceptives (34, 35). But here again, serum FSH level is low in these situations.

**Figure 1:**
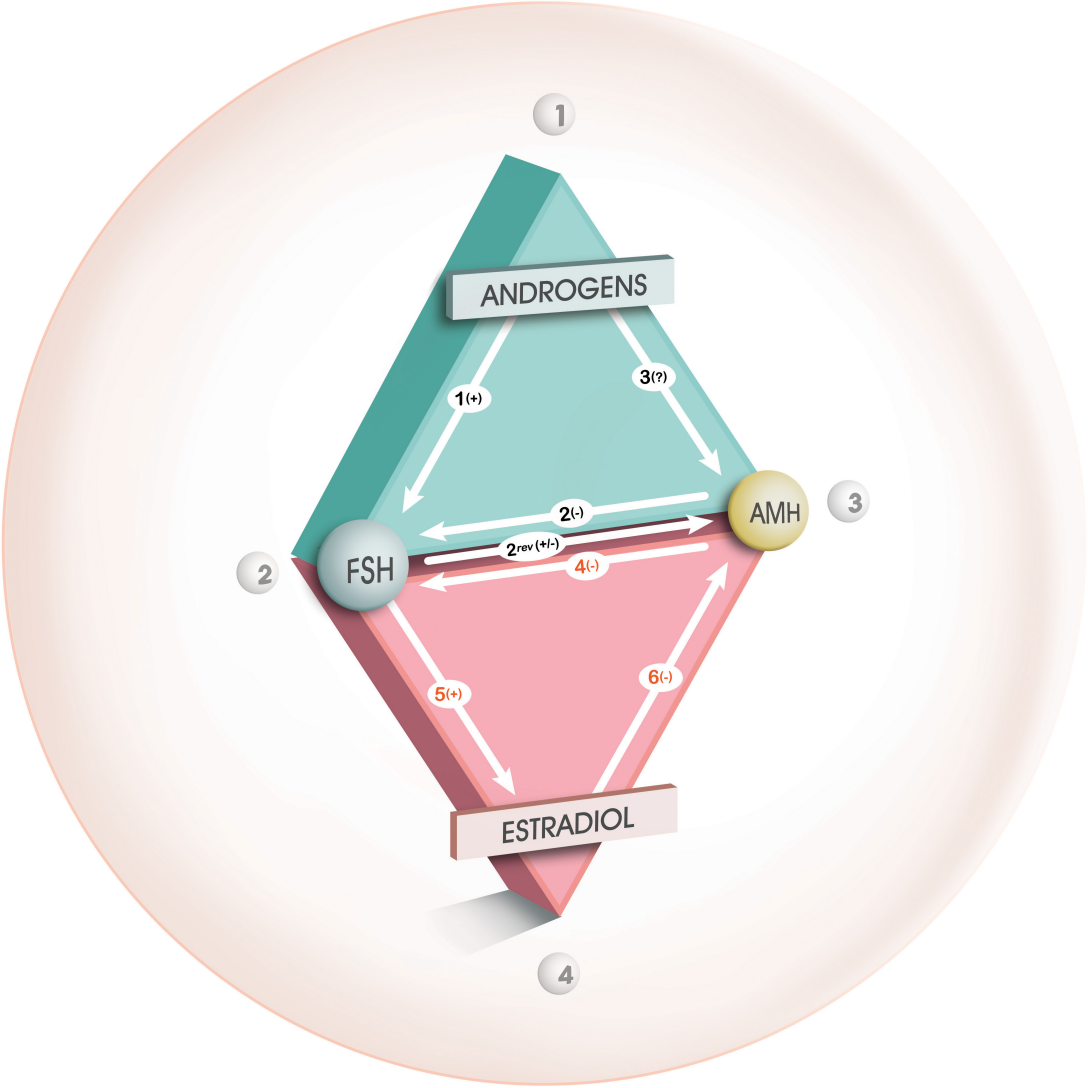
Interaction between androgens, FSH, AMH, and E2 during folliculogenesis. From Dewailly et al. (19), with permission. Relationships between androgens, FSH and AMH during the gonadotropin- independent follicular growth phase (green triangle) and between FSH, AMH and estradiol during the gonadotropin- dependent follicular growth phase (red triangle). “+,” “–,” or “?” indicate a positive, negative or uncertain effect, respectively, from one of the factors on the other. During the gonadotropin-independent follicular growth phase, the inhibitory effect of AMH mainly influences the promoting effect of FSH on follicular growth (arrow 2). According to our theory, FSH, whose receptors are enhanced by androgens (arrow 1), would stimulate the AMH production during this phase (arrow 2 rev), in the absence of estradiol. A direct effect from androgens on AMH production (arrow 3) is unlikely (see text for details). During the gonadotropin-dependent follicular growth phase, AMH is also involved in a triangular relationship with FSH and estradiol. During this phase, the inhibitory effect of AMH influences mainly the cell differentiation functions induced by FSH (arrow 4), in particular the induction of aromatase (arrow 5). This inhibitory effect will gradually subside, which will allow induction of aromatase by FSH, with consequent synthesis of estradiol which will in turn accelerates the extinction of AMH secretion in large antral follicles (arrow 6).

Similarly, *in vitro* experimental data are contradictory. An androgen-inhibitory effect of androgens on the secretion of AMH by Sertoli cells in men has been clearly demonstrated for many years (36). Crisosto et al. (37) demonstrated that high-dose testosterone was responsible for decreased levels of AMH expression in GCs from small bovine follicles. On the contrary, Zhang et al. (38) reported that testosterone caused an increase in AMH mRNA levels in GCs from mouse antral follicles. In women, some authors have not demonstrated any effect of 5α Dyhydrotestosterone (DHT) on the expression of AMH in GCs from control patients, whereas an increase was observed only in GCs from PCOS patients (39). Dilaver et al. (27) also observed this dose-dependent effect of DHT, while that of testosterone was either positive or null according to its concentration in the GCs culture medium. It should be noted that the contradictory results between these different studies on the effects of androgens on the expression of the AMH gene could be explained by the great variability of the models used (different animal species, cell type, analysis method). Moreover, the effect of androgens is to be seen in the complex interactions they have with other important actors at the GCs level, such as FSH and Estradiol (E2), which vary according to the follicular stage and which are not always taken into account in experimental studies (Figure 1) (19).

Several studies suggest an indirect effect of androgens, via an increase in the number of FSH receptors (FSHR) and/or estradiol receptors alpha (ERα). Many studies converge toward the promoting action of androgens on the transcription and translation of FSHR through genomic and non-genomic effects and this effect is likely enhanced in PCO [reviewed in (19)]. Consequently, the stimulating effect of FSH on AMH expression that occurs in small growing follicles from normal ovaries would be amplified in PCO (40). This can occur as long as follicles do not express aromatase as E2 inhibits AMH expression through its receptor ERβ (41, 42) (Figure 1).

This last phenomenon might be defective in GCs from PCO. Dilaver et al. (27) reported that excess androgens increase the ratio ERα/ERβ, resulting in increased AMH expression. The importance of the relative expression levels of ERα and ERβ has been shown earlier (43). Pierre et al. (39) recently reported a significant positive correlation between the ratio of ERα/ERβ transcripts and the concentration of AMH and an increase in the levels of ERα transcripts in cultured GCs from PCOS women. However, small growing follicles produce very little E2 and this effect of androgens through activation of ERα might not be relevant *in vivo*. Conversely, at the time of large antral follicle selection for dominance and when activation of ERβ is determinant, this effect of androgens maintaining AMH expression might be part of the mechanisms leading to the follicular arrest of PCOS (see below).

### Are the AMH Signaling Pathways Normal in the PCO GCs?

In addition to increased expression of AMH, the expression of AMH type 2 receptors (AMHR2) is amplified in PCO GCs (25, 39). Activation of AMHR2 results in a significant increase in phosphorylation of SMAD 1,5,8 in the mouse (44) and of SMAD 5 in luteinized human GCs (45). Intriguingly, Dilaver et al. (27) recently demonstrated in cultured GCs from PCOs a dose-dependent decrease in phosphorylation of SMAD 1,5,8 (P-SMAD 1,5,8) in the presence of AMH, while paradoxically the levels of transcripts of P-SMAD 1,5,8 was increased by about 50% in controls (but without reaching statistical significance). Obviously, if the implication of a deregulation of the AMH signaling pathways in PCOS seems to be an interesting issue, other subsequent studies are needed, especially concerning the involvement of inhibitory SMADs.

### What Are the Consequences of the Excess AMH on Ovarian Follicles, According to Their Stages?

#### Excess AMH Slows Initial Follicular Growth

This hypothesis is based on the seminal experiment of Durlinger et al. (46). The addition of AMH in cell culture media containing follicles from knockout mice for the AMH gene slowed follicular growth, even in the presence of FSH, suggesting an inhibitory effect of AMH on FSH-dependent proliferation of GCs.

In a situation of high AMH such as PCOS, a slowing of the initial “FSH-sensitive” follicular growth could thus occur and contribute to the accumulation of the number of growing follicles within the ovaries in these patients. However, few data specific to the human species have been found to confirm this pathophysiological hypothesis (3).

#### Excess AMH Decreases Apoptosis of GCs in Small Follicles

Some authors have suggested that AMH has an anti-atretic effect on growing follicles during initial follicular recruitment (27, 47). Some known pro-apoptotic agents, such as vitamin D and leptin, may act by decreasing the expression of AMHR2 and thus the anti-apoptosis effect of AMH on GCs (47).

The data available in the literature for PCOS women are relatively small. Webber et al. (48) demonstrated in cell culture models a lower apoptosis rate of GCs from pre-antral follicles in women with PCOS compared to controls. By immunocytochemistry, GCs from PCO are less and more frequently stained for the markers of apoptosis and anti-apoptosis than in controls, respectively (24, 49). High levels of AMH could be directly involved in this phenomenon, which would result in a “stock piling” effect (4) contributing to the excess number of growing follicles in the PCOs.

Finally, as menopause approaches, women with PCOS have significantly higher serum AMH levels than non-PCOS women in whom these levels are low or undetectable (50). This may explain why women with PCOS appear to reach menopause at a slightly later age than non-PCOS women (50).

#### Excess AMH Causes Follicular Arrest in Large Antral Follicles

This phenomenon results from complex interaction between AMH, aromatase, ERs and less likely LH (Figure 1).

AMH has been shown to significantly decrease not only FSHR expression but also ovarian aromatase expression [see (19)]. Physiologically, this protects small follicles from premature aromatase expression. When this protective effect of AMH exceeds its physiological role, because of its excess and/or because it lasts longer than it should, it could lead to a defect in the selection of the dominant follicle, causing what is called “follicular arrest.” The fact that AMH inhibits the FSH-dependent factors necessary for follicle dominance adds considerable importance to the elevated serum expression of AMH in PCOS and makes AMH an assumed central player in “follicular arrest.” In agreement, it has been shown that the emergence of a dominant follicle in anovulatory women with PCOS on recombinant FSH is preceded by a significant reduction in serum AMH level (51).

In addition, several authors have demonstrated premature expression of the LH receptor (LHR) in GCs of PCOS women. This has been suggested to be the cause of the arrest of follicular growth found in PCOS women with anovulation (52, 53). However, this hypothesis seems unlikely because other authors have more recently demonstrated a negative correlation between the concentration of AMH in the follicular fluid and the expression of the LHR in GCs (54).

#### The AMH Excess in Follicles Varies According to the PCOS Phenotype

This overexpression of AMH per follicle could vary depending on the PCOS phenotype. Thus, for some authors, in a population of PCOS women, the AMH/AFC ratio was significantly higher in patients with anovulation than in those with an ovulatory phenotype (phenotype C or asymptomatic ultrasound PCO) (22, 55). In contrast, other authors have shown higher AMH levels in hyperandrogenic PCOS women, regardless of ovulatory status (12, 29, 31). The question of variation in AMH expression according to the PCOS phenotype is in fact very complex because principal component analysis has shown that the markers of hyperandrogenism and oligoanovulation are closely related (30). However, when both hyperandrogenism and anovulation are statistically confronted with excess serum AMH, the association is significant with the latter, whereas the former would simply be a confounding factor (23).

To summarize, AMH excess in GCs from PCO would be an indirect consequence of hyperandrogenism and would be involved in the follicle excess of PCO and in the follicular arrest in anovulatory patients.

## Role of AMH In The Gonadotropin Dysregulation of PCOS

A high LH level is found in ~50% of women with PCOS, with a higher prevalence in women without metabolic impairment (56). It is secondary to the acceleration of the frequency of GnRH secretion which, for some authors, is thought to be the consequence of a negative feedback failure due to prenatal hypothalamic exposure to androgens (57). Conversely, mean FSH levels are lower than controls in many published series, with no precise explanation provided to date. Both phenomena lead to an increase in the LH/FSH ratio, which was used as a diagnostic criterion in the past, but was abandoned because it was too insensitive. AMH could be involved in this disturbance of gonadotropic function.

### There Is a Positive Link Between AMH and LH

In women with PCOS, serum levels of AMH and LH are positively correlated (7). This correlation has been shown to be independent of serum androgen and FSH levels (30, 51).

The causal relationship in this relationship has been the subject of debate. For some, the cause would be the high levels of LH that could stimulate AMH secretion and expression as shown by authors *in vitro* from luteinized GCs (23, 58). However, *in vivo*, GCs express LHR late, whereas AMH production begins in primary follicles and peaks before LHR expression (5). Alternatively, recent experimental data suggest that AMH is more likely to have extra-gonadal effects and in particular be capable of increasing the activity of GnRH neurons. The authors have shown that nearly 50% of GnRH neurons (murine and adult human) have specific receptors for AMH type 2 (AMHR2) (16). The combination of several *in vitro* and *in vivo* experiments showed that AMH increased the pulsatile secretion of GnRH-dependent LH through a central action. Indeed, electrophysiological experiments have revealed that exogenous AMH increased the neuronal activity of GnRH neurons; however, this could be an indirect action, as AMHR2 is very widely expressed in the hypothalamic regions, so a synergistic action of other cell types contributing to the increase in GnRH secretion cannot be excluded [for review see (59)]. Similarly, the authors demonstrated that *in vivo* administration of AMH (intracerebroventricularly) was accompanied by a dose-dependent increase in LH secretion and pulsatility. In the end, the increase in AMH concentration would lead to a chain reaction: hypothalamic neurons would start to secrete more GnRH, which would then increase the production and pulsatility of LH by the anterior pituitary gland.

AMH also appears to be able to exert its action at the pituitary level and regulate the activity of gonadotropic cells. It has recently been shown that the expression of the human and mouse AMHR2 gene in gonadotropic cells is regulated by GnRH (60). Indeed, using LβT2 cells, these authors showed that GnRH secreted at a high frequency (1 pulse/30 min) increased AMHR2 expression by gonadotropic cells while a lower frequency (1 pulse/2 h) was without effect. However, the implication of the regulation of pituitary AMHR2 expression as a function of GnRH pulsatility remains to be elucidated in humans and especially in PCOS.

These results raise the hypothesis that the extra-gonadal action of AMH could either be at the origin of, or contribute to, the vicious circle of neuroendocrine and gonadal dysregulation encountered in PCOS.

### The Negative Link Between AMH and FSH: A Complex Issue

Low to normal serum FSH levels have long been reported in PCOS (61), even after adjustment for BMI and the number of 2–9 mm follicles (13). Several studies have reported a negative relationship between serum FSH and AMH levels (8, 51) but no clear explanation has been provided so far. It is unlikely that this reflects a negative effect of FSH on AMH production. In fact, the opposite is suggested by situations of congenital gonadotropic insufficiency where AMH level is decreased and increases under exogenous FSH (62). These contradictory data illustrate the complex relationships between AMH and FSH that may operate at the ovarian and/or pituitary-hypothalamic levels and which vary according to disease state. In the case of PCOS, we hypothesize that by accelerating the pulse frequency of GnRH (see above), an excessive AMH level would increase pituitary secretion of LH to the detriment of FSH (63). It is clear that more attention needs be paid to this issue.

To summarize, new experimental data suggests that AMH is involved in the neuro-endocrine deregulation of PCOS but no human data is available so far to confirm this hypothesis.

## Is Excess AMH Involved In The Trans-Generational Transmission of PCOS?

It was in the early 2000s that the hypothesis of prenatal programming of PCOS in relation to gestational hyperandrogenism was first suggested (64). Following this discovery, numerous studies confirmed in various animal models that high testosterone levels during gestation could lead to the appearance of a PCOS phenotype in the offspring (mouse, ewe and non-human primate models) [for review see (57, 65)]. In women with PCOS, the hypothesis of androgen-related prenatal programming is supported by a whole series of studies [see (17) for review], but the origin of this gestational hyperandrogenism remains unknown so far.

Recent studies suggest that AMH may be involved in this phenomenon. Circulating AMH levels are higher in pregnant women with PCOS compared to those with normal fertility (66, 67) and are correlated with androgen levels (67). These results therefore suggest that AMH at relatively high concentration during pregnancy could itself be the cause of prenatal programming of PCOS. This has recently been tested experimentally (66). The authors demonstrated that injection of the bioactive form of AMH (AMHc) into late gestation mice was responsible for the appearance of a hyperandrogenic PCOS phenotype in the offspring in adulthood. In this model, called PAMH, high AMH concentrations during gestation resulted in increased pulsatility of GnRH and LH, which was responsible for gestational hyperandrogenism. Excess maternal LH alone or in combination with AMH would also lead to a decrease in placental aromatase, increasing maternal bioavailable testosterone and causing fetal exposure to androgen excess. This would induce a cascade of events in the offspring leading to an increase in hypothalamic neuronal excitability. In adult offspring, mice show an increase in excitatory afferents responsible for an increase in the excitability of GnRH neurons. The hyperactivity of GnRH neurons then stimulates ovarian steroidogenesis and participates in the vicious circle observed in PCOS by reducing the negative feedback of E2 and progesterone on LH. Prenatal treatment with a GnRH antagonist in PAMH mice prevents the occurrence of the disorders previously observed in the offspring (66). The authors thus demonstrated the predominant role of GnRH, via AMH, in the *in utero* programming phenomenon responsible for the neuroendocrine abnormalities characteristic of PCOS appearing in the offspring.

Finally, it should be noted that this new PAMH mouse model suggests that the maternal hyperandrogenisation observed in PCOS is the result of a central action of AMH on GnRH (and LH) contributing to an increase in ovarian steroidogenesis and an inhibition of placental aromatase expression, leading to an increase in testosterone bioavailability (66). In agreement, continuous administration of a P450 Aromatase Inhibitor induces Polycystic Ovary Syndrome with a metabolic and endocrine phenotype in female rats at adult age (68). In women, inhibition of placental aromatase expression may be the main mechanism in the *in utero* programming of PCOS as serum maternal androgen and LH levels are not as high as in PAMH mice. A decrease in placental aromatase has effectively been observed in women with PCOS who have given birth (69). Studies conducted in mice therefore offer interesting new perspectives that will have to be confirmed in the future by clinical studies in women, since the mouse model is poly-ovulatory and is not perfectly superimposable on the human condition.

To summarize, maternal AMH excess might be one of the causes of *in utero* programming of PCOS, at least in a subset of patients.

## Conclusion

There is still much knowledge to be acquired to fully understand the pathophysiological role played by the AMH in the PCOS. Clearly, the autocrine action of excess AMH within the GCs is probably the main element of its involvement in the folliculogenesis and anovulation disorder. However, the recent discovery of its endocrine action of retrocontrol on the hypothalamus and the placenta opens up avenues of research likely to lead to new curative or even preventive treatments. For instance, when they are available, antagonists of the AMHR2 might prove to be able to lessen the LH and follicle excess and thus to improve the emergence of a dominant follicle and increase the chances of pregnancy without any ovarian stimulation.

## Author Contributions

DD contributed to review design, execution, acquisition, analysis and interpretation of data, manuscript drafting, and critical discussion. A-LB, GR, AD, and SC-J contributed to acquisition and interpretation of data, manuscript drafting, and critical discussion. All authors read and approved the final manuscript.

## Conflict of Interest

The authors declare that the research was conducted in the absence of any commercial or financial relationships that could be construed as a potential conflict of interest.
